# Paliperidone induced neutropenia in first episode psychosis: a case report

**DOI:** 10.1186/s12888-021-03073-w

**Published:** 2021-02-06

**Authors:** Natalie Martos, William Hall, Alicia Marhefka, Thomas W. Sedlak, Frederick C. Nucifora

**Affiliations:** Deptments of Psychiatry and Behavioral Sciences, Johns Hopkins University School of Medicine, 600 N. Wolfe Street, Baltimore, MD 21287 USA

**Keywords:** First episode psychosis, Neutropenia, Paliperidone, Schizophrenia, Clozapine

## Abstract

**Background:**

Neutropenia, a decrease in total number of neutrophils below 1500/mm^3^ and particularly severe neutropenia, defined as neutrophils less than 500/mm^3^, is a potential adverse effect of antipsychotic medications that can lead to increased risk of infections and death. However, much of the attention on the potential adverse effect is centered exclusively on clozapine, which remains the only antipsychotic medication in the United States requiring standardized monitoring of blood work. We demonstrate here that paliperidone can also cause neutropenia and therefore clinicians should be aware of this possibility especially during initiation of treatment.

**Case presentation:**

The following report presents the case of a 23-year-old African American male with first episode psychosis who developed neutropenia after initiation of paliperidone. Neutropenia resolved after discontinuation of paliperidone and initiation of an alternative antipsychotic, haloperidol.

**Conclusions:**

This case report demonstrates an example of paliperidone induced neutropenia which resolved with a switch to haloperidol. We conclude that when initiating paliperidone, clinicians should be more aware of the risk of neutropenia. Moreover, neutropenia may be a more common and overlooked issue in patients on antipsychotic medications other than clozapine and increased awareness of comparative risk across antipsychotics could help direct treatment.

## Background

Neutropenia, a decrease in total number of neutrophils below 1500/mm^3^, is a potential adverse effect of antipsychotic medications that can lead to increased risk of infections. However, much of the attention on the potential for neutropenia and severe neutropenia, defined as neutrophils less than 500/mm^3^, is centered exclusively on clozapine, which remains the only antipsychotic medication in the United States requiring standardized monitoring of blood work [1–3].

In the following case report, we describe a relatively treatment-naïve patient with new onset psychosis whose absolute neutrophil count (ANC) dropped significantly with the initiation of the atypical antipsychotic, paliperidone. We later discuss the future implications of neutropenia in antipsychotic medications other than clozapine.

## Case presentation

A 23-year-old African American male with previous high level of social and academic functioning, family history of depression, and no past medical or psychiatric history was admitted to an inpatient psychiatric unit for treatment of several months of uncharacteristic, odd behavior and disorganized thought process. On presentation, the patient could not provide a coherent narrative. His family and friends reported gradual onset of odd behavior within the year prior to admission. These behaviors included wearing his rugby helmet around his college campus, discarding clothes in the trash, and becoming increasingly disheveled in appearance. He drove across the state, in the middle of the night, to his mother’s home to then fearfully claim he was being pursued and his mother was trying to kill him.

Several weeks prior to admission, the patient was hospitalized, diagnosed with schizophrenia, and prescribed perphenazine 8 mg twice daily and doxepin 50 mg nightly. After 7 days of inpatient care he was discharged. His disorganized behavior returned 3 days after discharge, prompting his mother to bring him for inpatient psychiatric admission.

On presentation, he was responding to internal stimuli, regularly glancing around the room during the interview. His speech was latent with intermittent thought blocking. He reported somatic delusions regarding his heart and a general delusional atmosphere feeling that everything around him was connected. He reported an auditory hallucination of the devil’s voice.

Admission labs (WBC 5300, ANC 2100), urine toxicity, and head CT were all unremarkable. He was diagnosed with schizophrenia and started on aripiprazole 10 mg, which was increased to 20 mg. He was also treated with escitalopram 10 mg daily given concern for possible affective component and clonazepam 0.25 mg twice daily for anxiety. After nearly 2 weeks, the patient demonstrated little improvement on mental status exam.

Given lack of response to aripiprazole, he was transitioned to oral paliperidone 6 mg. His ANC prior to paliperidone initiation was 4120. Following 15 days of paliperidone 6 mg, he started to respond with improvement in his psychotic symptoms. However, his complete blood count at day fifteen of paliperidone revealed an ANC of 1210 with a nadir of 960 (WBC nadir of 3720) on serial monitoring. Aside from neutropenia, there were no other lab abnormalities. Peripheral smear showed mild leucopenia with neutropenia and relative lymphocytosis. Antinuclear antibody (ANA) and human immunodeficiency virus (HIV) were both negative. Physical exam was unremarkable. Hematology specialists concluded that the timeline and negative work-up were most consistent with drug-induced neutropenia. Paliperidone was discontinued after 20 days of treatment and haloperidol 10 mg nightly was started. ANC was monitored three times weekly. Gradually, his counts improved and 16 days after discontinuation of paliperidone, returned to normal with an ANC of 2070 (WBC 5220). While on haloperidol, the patient showed progressive improvement of his symptoms. By discharge he demonstrated spontaneity of speech and greatly improved content and organization of thoughts.

## Discussion and conclusions

In our case, neutropenia developed after initiation of paliperidone and resolved within 16 days of discontinuation. Neutrophil levels remained normal after the initiation of haloperidol, indicating that neutropenia was most likely related to the initiation of paliperidone.

We also considered other contributing factors to his neutropenia. He was also treated with clonazepam and escitalopram during his course but with no evidence of temporal relationship to his neutropenia. However, there are reports of clonazepam induced neutropenia [4]. Though clonazepam had been discontinued by the time the patient developed neutropenia, we cannot rule out a possible synergistic effect from both paliperidone and clonazepam. Additionally, while there are no agreed upon risk factors for antipsychotic induced neutropenia (particularly paliperidone-induced), one might reasonably apply risk factors for clozapine induced neutropenia to this case. And in this case, neuroleptic naivety, higher doses of neuroleptics, African American race, male gender, and younger age all may have placed him at greater risk of developing neuroleptic induced neutropenia [5, 6]. Furthermore, we considered the role of benign ethnic neutropenia (BEN); however, hematology consultants did not consider this a factor given his ANC counts being consistently greater than 1500 prior to initiation of neuroleptics.

To date, we are only aware of three other case reports on neutropenia induced by paliperidone. In all three cases, neutropenia resolved within several days to weeks after discontinuation of paliperidone [7–9]. In one case, lithium was successfully added temporarily to counteract the neutropenia [7]. Two of the reports suggested that neutropenia was dose related and concomitant psychiatric medications (divalproex sodium and quetiapine in one case, risperidone in the other) could have also synergistically contributed to development of neutropenia [7, 8]. In all cases, patients had been on their previous regimens without neutropenia prior to initiation of paliperidone. The patient described here highlights that the decrease in ANC may occur at low to middle doses of paliperidone since the Food and Drug Administration FDA maximum dosage is 12 mg and early in the course of treatment. Of note, paliperidone (9-hydroxyrisperidone) is a metabolite of risperidone. A literature review demonstrates evidence of risperidone induced blood dyscrasias, including neutropenia [10, 11]. Thus, it stands to reason that risperidone’s metabolite could also lead to neutropenia.

Our case report highlights the potential for antipsychotics other than clozapine to cause neutropenia. Although clozapine is the antipsychotic most commonly associated with agranulocytosis or severe neutropenia (term now used by the Clozapine Risk Evaluation and Mitigation Strategies program) with an estimated risk of approximately 0.68%, other antipsychotics have also been linked to neutropenia and severe neutropenia including the majority of second generation antipsychotics [12–14]. We now present evidence for paliperidone causing neutropenia in a case of first episode psychosis (occurring within the first year of treatment). While there are no guidelines for routine ANC monitoring when using any antipsychotic other than clozapine, our case suggests the potential risk of neutropenia when initiating paliperidone. If we had failed to recognize the precipitous fall in our patient’s neutrophil count, he may have remained on paliperidone indefinitely with increased risk for medication-related morbidity and mortality.

Currently, clozapine is widely recognized as one of the most effective antipsychotic medications available but is underutilized largely due to the barriers to prescribers and heavy burden placed on patients with required regular blood monitoring [2]. After many years of implementation, the utility of long-term clozapine monitoring is still debated. Some evidence suggests that there is not a significant increase in risk for developing neutropenia while on clozapine compared to other medications [12–15] and following the ANC after the first 6 months of treatment may not be as beneficial in preventing fatalities as previously thought [12, 15]. Based on the experiences with clozapine, it would likely be counterproductive to spread this monitoring burden to even more antipsychotics, such as paliperidone, in efforts to mitigate this side effect risk. Further evaluation of how high the risk of neutropenia and severe neutropenia is in the population treated with paliperidone, as well as other newer antipsychotics, would be necessary to fully appreciate the true risk. Ultimately, striking a balance between safely prescribing antipsychotics while still providing effective and accessible treatment will be crucial in the future as newer medications like paliperidone become more widely used. However, this case report highlights the importance of clinicians being aware of the risk and considering obtaining routine ANCs when initiating paliperidone and other antipsychotics.

This case report demonstrates an example of paliperidone induced neutropenia which resolved with a switch to haloperidol. In general, we conclude that there is sufficient and mounting evidence that most antipsychotics can cause neutropenia and that there may be value in monitoring ANCs early in the course of treatment to ensure stability. However, we would caution against mandatory monitoring with a national registry as this has contributed to the underutilization of clozapine, and thus could disrupt effective utilization of antipsychotics in general. This could also help make the argument to relax mandatory monitoring of clozapine since neutropenia may not be specific to clozapine. The hope is that this case will add to clinicians’ awareness that neutropenia is a risk with neuroleptic treatment, and that it will also spur further studies regarding comparative risk for neutropenia among all neuroleptics and a re-thinking of a mandatory registry for clozapine.

## Data Availability

Data sharing is not applicable to this article as no datasets were generated or analyzed during the current study.

## Abbreviations

ANC: Absolute Neutrophil Count
ANA: Antinuclear Antibody
HIV: Human Immunodeficiency Virus
FDA: Food and Drug Administration
